# Comparative Evaluation of Decellularized Human Amniotic Membrane and Wharton’s Jelly in a Rat Model of Myocardial Infarction: Experimental Study

**DOI:** 10.3390/cimb48060579

**Published:** 2026-06-01

**Authors:** Marcos Antônio Denk, Isabella Cristina Mendes Rossa, Luize Kremer Gamba, Anna Clara Faidiga Silva, Julia Letícia de Bortolo, Paulo Cesar Lock Silveira, Camila da Costa, Júlio Cesar Francisco, Luiz César Guarita-Souza

**Affiliations:** 1 Graduate Program in Health Sciences, School of Medicine, Pontifícia Universidade Católica do Paraná (PUCPR), Curitiba 80215-901, Paraná, Brazil; isabella.rossa@pucpr.edu.br (I.C.M.R.); luizekremer@gmail.com (L.K.G.); annafaidiga@hotmail.com (A.C.F.S.); jldbortolo@gmail.com (J.L.d.B.); julio.francisco1@pucpr.br (J.C.F.); cesar.souza@pucpr.br (L.C.G.-S.); 2 Laboratory of Experimental Physiopathology, Program of postgraduate in Science of Health, Universidade do Extremo Sul Catarinense, Santa Catarina 88806-000, Santa Catarina, Brazil; psilveira@unesc.net (P.C.L.S.); camiladacosta49@gmail.com (C.d.C.); 3 Postgraduate Program in Biomedical Engineering (PPGEB-CT), Universidade Tecnológico Federal do Paraná, Curitiba 81310-900, Paraná, Brazil

**Keywords:** myocardial infarction, heart failure, amniotic membrane, Wharton′s jelly, decellularization, myocardial remodeling

## Abstract

**Background/Objectives:** Acute myocardial infarction (AMI) remains a major cause of global morbidity and mortality and is a leading factor in the development of heart failure. This study investigated the regenerative potential of decellularized human amniotic membrane (HAM) and Wharton’s jelly (WJ) in a rat model of left ventricular dysfunction induced by acute myocardial infarction (AMI). **Methods:** Twenty-three rats underwent left anterior descending coronary artery ligation and were randomized into three groups: control (saline), WJ (decellularized WJ), and HAM (decellularized HAM). **Results:** After 30 days, echocardiographic, histopathological, and immunohistochemical assessments were performed. No significant differences in ventricular function were observed among groups. However, the HAM-treated group showed a significant reduction in myocardial fibrosis compared with the control (*p* = 0.009), suggesting attenuation of post-infarction remodeling. Despite the absence of measurable functional recovery, HAM demonstrated potential to promote more favorable tissue organization. Study limitations include the lack of a sham-operated group, short follow-up period, and absence of quantitative decellularization validation. **Conclusions:** Overall, the results indicate that decellularized HAM may act as a structural modulator of myocardial remodeling, warranting further studies with longer follow-up and combination approaches, such as cell-based or growth factor-enhanced therapies.

## 1. Introduction

Acute myocardial infarction (AMI) remains one of the major causes of morbidity and mortality worldwide and represents a key contributor to the development of heart failure (HF). The progressive loss of cardiomyocytes following ischemic injury leads to extensive ventricular remodeling and contractile dysfunction, resulting in irreversible cardiac damage [1,2]. Although advances in reperfusion therapy and pharmacological interventions have improved survival, many patients still experience chronic ventricular dysfunction, underscoring the urgent need for new therapeutic strategies that promote myocardial regeneration [3,4]. Heart transplantation remains the gold standard for end-stage HF, but the scarcity of donors and the lifelong risks associated with immunosuppression highlight the importance of developing alternative regenerative approaches [5]. In this context, tissue engineering and regenerative medicine offer promising pathways, particularly using extracellular matrix (ECM)-based biomaterials capable of supporting cell adhesion, migration, and differentiation [6,7].

Among ECM-derived biomaterials, the human amniotic membrane (HAM) and Wharton’s jelly (WJ) have gained attention due to their rich composition in collagen, glycosaminoglycans, and growth factors that contribute to tissue repair [8,9]. HAM has demonstrated anti-inflammatory and antifibrotic properties and has been used clinically for decades in ophthalmology and wound healing [10]. WJ, the gelatinous matrix surrounding the umbilical cord vessels, is rich in type III collagen and hyaluronic acid, and its acellular form has been investigated for promoting angiogenesis and improving tissue integration [11,12].

The decellularization process, which removes cellular components while preserving ECM integrity, aims to minimize residual immunogenicity and improve biocompatibility, even though perinatal tissues such as HAM and WJ are naturally low in immune reactivity [13]. Importantly, decellularization provides a uniform scaffold that facilitates reproducible experimental results and serves as a platform for future incorporation of cells or bioactive factors [14,15]. Recent studies have highlighted that combining decellularized scaffolds with mesenchymal stem cells or angiogenic factors can enhance cardiac repair by promoting neovascularization, reducing fibrosis, and improving contractile function [16,17].

However, evidence regarding the isolated effects of decellularized HAM and WJ on myocardial remodeling remains limited, particularly in models of established left ventricular dysfunction. Given these considerations, the present study aimed to compare the effects of decellularized HAM and WJ implants on myocardial fibrosis and ventricular function in a rat model of post-infarction left ventricular dysfunction.

We hypothesized that both biomaterials could modulate fibrosis and support structural remodeling, even in the absence of exogenous cells, thereby providing foundational evidence for future combined regenerative strategies.

## 2. Materials and Methods

### 2.1. Study Protocol

The Animal Use Ethics Committee (CEUA) of the Pontifical Catholic University of Paraná (PUCPR), under protocol number 02285, approved this study. This experimental study was conducted in accordance with the guidelines of the Brazilian College of Animal Experimentation [18]. A total of 70 adult male Wistar rats (250–300 g), aged 2–3 months, were used and maintained under controlled conditions of temperature (22 ± 2 °C), humidity (50–60%), and a 12-h light–dark cycle. The animals had free access to water and standard chow. They were subjected to surgical induction of acute myocardial infarction (AMI) by ligation of the left anterior descending (LAD) coronary artery to produce left ventricular dysfunction (LVEF ≤ 45%).

**Figure 1:**
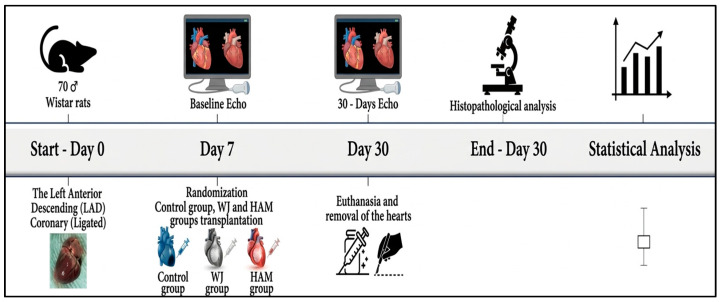
Schematic representation of this study. Myocardial infarction progression across the three groups. Scaffold implantation procedure in the control group, WJ group, and HAM group.

Seven days after AMI induction, a blinded investigator performed a transthoracic echocardiography and recorded the data independently from the research team. The experimental units confirmed with left ventricular dysfunction were randomized into three experimental groups. Every effort was made to minimize animal suffering and to use the fewest necessary animals to achieve statistically significant results. The overall flow of animal inclusion, exclusions, and mortality throughout the experimental protocol is summarized in (Figure 1).

These animals were randomly and evenly assigned to three experimental groups:

- **Group I (Control):** Injection of sterile saline solution (0.9% NaCl) into the infarcted myocardial region.
- **Group II (Wharton’s jelly):** Implantation of decellularized human Wharton’s jelly patch into the infarcted myocardial region.
- **Group III (Amniotic membrane):** Implantation of decellularized human amniotic membrane into the infarcted myocardial region.

After randomization, the experimental units belonging to the same group were housed in the same cage, which was properly labeled, and individual identification tags were affixed to the tails of the same group. On the 30th day, all surviving animals underwent a second transthoracic echocardiogram assessment performed by a blinded investigator to measure left ventricular ejection fraction (LVEF), end-systolic volume (ESV), and end-diastolic volume (EDV) and were subsequently euthanized. Myocardial tissue samples were collected for histopathological and immunohistochemical analysis.

### 2.2. Acquisition of Acellular Amniotic Membrane (HAM) and Wharton’s Jelly (WJ)

Human umbilical cords and placentas were obtained from two consenting mothers (gestational age: 36–40 weeks) who signed informed consent forms in accordance with a protocol approved by the Research Ethics Committee of Pequeno Príncipe Hospital (approval numbers 659.204/2014 and 0948-11). HAM and WJ were collected under sterile conditions immediately after placental expulsion in the surgical setting.

The collected material included segments of the umbilical cord, chorionic plate, and chorionic villi. All processing procedures, including membrane decellularization, mesenchymal stem cell isolation, culture, and phenotypic characterization, were performed at the Tissue Regeneration Center of PUCPR. All donors tested negative for HIV, hepatitis B and C, and syphilis. Both WJ and HAM were processed under aseptic conditions. Tissues were washed in phosphate-buffered saline (PBS) containing antibiotics (100 U/mL penicillin, 100 µg/mL streptomycin) and treated with 0.01% sodium dodecyl sulfate (SDS) and 0.01% sodium deoxycholate (SD) at 37 °C under agitation for 24 h, followed by triple PBS rinsing [11].

The decellularization process aimed to remove cellular components while preserving ECM architecture. Histological evaluation of representative samples demonstrated an average thickness of approximately 30 µm (range 20–40 µm) after decellularization. The membrane preserved its basement membrane and stromal matrix architecture, providing structural support for its use as an epicardial patch.

Biomaterial implantation was performed seven days after acute myocardial infarction (AMI), following echocardiographic confirmation of left ventricular dysfunction (EF ≤ 45%). This point was selected to target the early remodeling phase, characterized by the resolution of inflammation and the initiation of fibrotic deposition. This provides a more stable microenvironment for graft integration and retention [19].

### 2.3. Experimental Induction of Acute Myocardial Infarction (MI)

**Figure 2:**
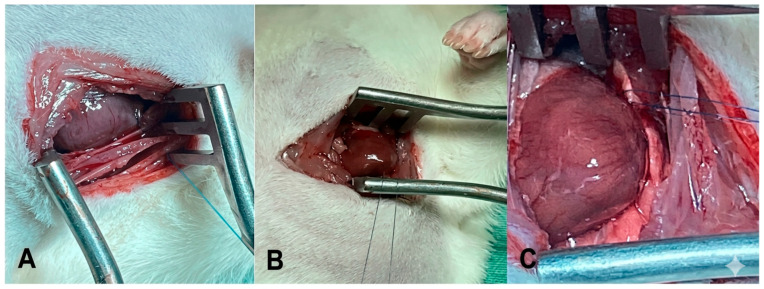
(**A**) Lateral thoracotomy for LAD access in an animal model; (**B**,**C**) Myocardial ischemia resulting from ligation of the LAD.

After each animal was anesthetized with ketamine (50 mg/kg, IM) and xylazine (10 mg/kg, IM) and orotracheally intubated, the animal was connected to a mechanical ventilator (HARVARD^®^, Inc., respirator model 683, Holliston, MA, USA) using room air (21% O_2_). The chest was prepared aseptically with povidone-iodine. A left lateral thoracotomy was performed in the third intercostal space; the left pleura was opened and the pericardium incised to optimally expose the heart. The left atrium was retracted to locate the left coronary artery (LAD) between the pulmonary artery and the left atrium. (Figure 2A).

The heart was exteriorized, and the left atrium was mobilized to expose the left coronary artery, which was ligated with a 7-0 monofilament Prolene suture between the pulmonary artery outflow tract and the left atrium. Successful infarction was confirmed by the color change of the infarcted region (Figure 2B,C). The heart was then returned to the thorax, the lungs were reinflated, and the chest was closed in layers using a 4.0 non-absorbable monofilament nylon suture according to established protocols [20,21].

The operations and all analyses were performed blinded. The animals were kept in cages and received commercial feed and water ad libitum following recovery from anesthesia; the animals were housed in standard cages under controlled environmental conditions, with a 12-h light/dark cycle, and were given free access to food and water.

### 2.4. Transthoracic Echocardiography (7th Day)

Echocardiographic evaluations were performed 7 days post-MI (and repeated at 30 days post-biomaterial implantation). Animals were lightly anesthetized via intramuscular injection of ketamine (50 mg/kg) and xylazine (10 mg/kg) and examined using a Hewlett Packard Sonos 5500 echocardiograph (Philips Medical Systems, Andover, MA, USA) with an S12 sector probe (5–10 MHz). Measurements of end-systolic and end-diastolic areas, left ventricular lengths, and heart rate were used to calculate the left ventricular ejection fraction (LVEF), end-systolic volume (ESV), and end-diastolic volume (EDV) using Simpson’s method [22]. The same blinded cardiologist performed all evaluations. Only animals with an LVEF ≤ 45% were included to characterize ventricular dysfunction; those with LVEF > 45% (approximately 30% of the animals) were excluded and euthanized.

### 2.5. Biomaterial Implantation Procedure

Seven days after myocardial infarction (MI), 36 surviving animals were randomized into three groups. All implants (CG, WJG, and HMAG) were administered following the functional confirmation of ventricular dysfunction, rather than at an earlier time point. This subacute phase corresponds to an active remodeling window characterized by persistent inflammatory signaling and progressive extracellular matrix reorganization, which allows for the modulation of fibrosis and ventricular geometry.

The procedure was carried out on 36 animals considered eligible for the study (i.e., those with an LVEF of ≤45%). Anaesthesia was administered using 5% ketamine (Vetanarcol^®^, König do Brasil Ltd.a., Santana de Parnaiba, Brazil) at a dose of 50 mg/kg in conjunction with 2% xylazine hydrochloride (Rompun^®^, Bayer S.A., São Paulo, Brazil) at a dose of 10 mg/kg. After the chest was prepared with topical povidone–iodine antisepsis, a median sternotomy was performed, and the animals were reconnected to the mechanical ventilation system. Small animals were ventilated with volume respirators (Harvard^®^, model 683, Holliston, MA, USA) with 21% oxygen (room air).

For myocardial implantation, WJG and HAMG patches were prepared as single-layer amniotic membranes with an approximate thickness of 80–120 µm, as determined in representative histological sections. After decellularization and storage, square grafts measuring 7 × 7 mm^2^ were trimmed from the amniotic sheet to preserve the native basement membrane and stromal architecture.

**Figure 3:**
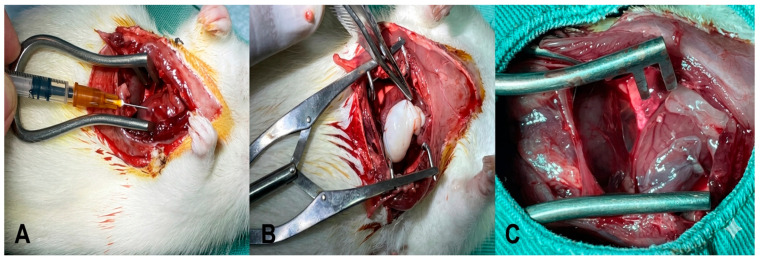
(**A**) Control group (CG) intramyocardial injection of 0.9% saline solution (n = 12), (**B**) Wharton’s jelly group (WJG) implantation of decellularized Wharton’s jelly patch (n = 12); (**C**) Human amniotic membrane group (HAMG) implantation of decellularized human amniotic membrane patch (n = 12).

**Group 1 (n = 12)—Control group saline solution (0.9% NaCl):** three intramyocardial injections were made into the infarcted area with sterile saline solution (0.9% NaCl). After reviewing hemostasis and insufflating the lungs, the chest wall was sutured in planes with 4.0 non-absorbable monofilament mononylon suture (Figure 3A).

**Group 2 (n = 12)—WJ implantation:** a 7 × 7 mm^2^ segment of the WJ was implanted over the ischemic area. The grafts were fixed using Prolene 6.0 sutures. The chest wall was sutured in planes with a non-absorbable monofilament Prolene suture (Figure 3B). After reviewing hemostasis and insufflating the lungs, the chest wall was sutured in planes with 4.0 non-absorbable monofilament mononylon suture.

**Group 3 (n = 12)—HAM implantation:** a 7 × 7 mm^2^ segment of the HAM was implanted over the ischemic area. The grafts were fixed using Prolene 6.0 sutures. The chest wall was sutured in planes with a non-absorbable monofilament Prolene suture (Figure 3C). After reviewing hemostasis and insufflating the lungs, the chest wall was sutured in planes with 4.0 non-absorbable monofilament mononylon suture.

### 2.6. Transthoracic Echocardiography (30th Day)

On the 30th day after coronary occlusion, 23 surviving animals underwent a second echocardiographic evaluation under the same conditions as on the 7th day. Two-dimensional transthoracic echocardiography was performed using an ultrasound imaging system equipped with S12 (5–12 MHz) and 15L6 (7–15 MHz) sector transducers, specifically designed for small-animal imaging and capable of frame rates up to 160 Hz.

Images were acquired in two-dimensional B-mode using parasternal long-axis and short-axis views. Left ventricular (LV) volumes and ejection fraction were calculated using the modified Simpson’s method from apical-equivalent views adapted for small-animal imaging.

The following parameters were obtained: LV end-diastolic surface area, LV end-systolic surface area, LV end-diastolic length, LV end-systolic length, and heart rate. These measurements were used to calculate end-diastolic volume (DV, mL), end-systolic volume (SV, mL), and left ventricular ejection fraction (LVEF). All measurements were obtained from three consecutive cardiac cycles and averaged for analysis. All analyses were performed by the same experienced cardiologist, blinded to group allocation and study phase. Each parameter was measured three times, and the mean value was used for statistical analysis. This methodology has been widely applied and validated in experimental rat models of myocardial infarction, providing reliable functional assessment under standardized acquisition conditions.

### 2.7. Euthanasia

All of the animals were euthanized with intravenous sodium pentobarbital at a dose of 200 to 250 mg/kg. With a view to animal welfare, if there were clinical manifestations of the disease, such as signs of respiratory depression, abnormal breathing, wheezing, and nasal secretion; acute lung edema; factors associated with cardiac pump failure, such as decreased peripheral circulation and blue and cold extremities; jaundice; and secretions in the eyes, the animals would be euthanized before the period stipulated by the research.

### 2.8. Histological and Immunohistochemical Analysis

At the conclusion of the study, the hearts were harvested 30 days post-implantation. For the immunohistochemical analysis, 4 µm paraffin sections were placed on salinized slides and cleared in xylene before being rehydrated through a series of decreasing alcohol concentrations. Antigen retrieval was achieved via heat treatment in citrate buffer (pH 6.0) for α-actin, desmin, and sarcomeric actin or in EDTA buffer (pH 9.0) for CD31 and CD68. The slides were then cooled to room temperature.

Endogenous peroxidase was quenched using hydrogen peroxide and non-specific reactions were reduced with normal serum or bovine serum albumin. The following primary antibodies were used: α-smooth muscle actin (mouse monoclonal, clone 1A4, Abcam, Cambridge, UK; ab7817, 1:100), desmin (mouse monoclonal, Abcam, ab8592, 1:100), CD31 (rabbit polyclonal, Abcam, ab9498, 1:50), CD68 (mouse monoclonal, Abcam, ab283654, 1:50), and sarcomeric actin (mouse monoclonal, Abcam, ab68168, 1:200). The sections were incubated overnight at 4 °C, followed by polymer-based HRP detection, DAB chromogen development, and hematoxylin counterstaining.

Positive control tissues were processed in parallel and negative controls were generated by omitting the primary antibody or substituting it with isotype-matched immunoglobulin. Slides were evaluated under light microscopy by five blinded observers and quantitative analysis was performed in ten representative fields per sample, using standardized image parameters, following the protocol described by Rahman et al. [23].

### 2.9. Collagen Quantification

Histological sections (5 µm thick) were stained with H&E for nuclear and extracellular matrix characterization and with Picrosirius Red for specifically identifying type I and III collagen fibers. The middle layer of the left ventricular sections was hydrated with 0.5% Sirius Red in aqueous saturated picric acid (Sigma-Aldrich, St. Louis, MO, USA) and examined under polarized light using a Zeiss Axiovert S100 TV microscope (Carl Zeiss, Jena, Germany). Ten non-consecutive fields were randomly selected in each heart region. The collagen volume was quantified as the positive Sirius Red area relative to the total connective tissue area, as described by [7].

### 2.10. Infarct Area

The infarct area was estimated on the same sections used for collagen quantification but stained with Gomori’s trichrome. Digital images were analyzed with Zen2 Blue Edition software (Carl Zeiss Microscopy GmbH, Jena, Germany; Version 3.4.91.00000) to delineate the infarcted area by comparing the fibrotic region to the total endocardial length. Measurements were performed in triplicate by a blinded observer, and the mean value was calculated.

### 2.11. Statistical Analysis

The results for the EF (%), SV (mL), and DV (mL) variables were summarized using the mean, standard deviation, median, minimum, and maximum. An analysis of variance (ANOVA) model with one factor was used to compare the groups in terms of the day 7 assessment results and the differences between the day 7 and day 30 assessments in terms of the ejection fraction, systolic volume, and diastolic volume variables. The one-factor analysis of covariance (ANCOVA) model was used to compare the groups regarding the results on day 30, including the results of the 7-day assessment as a covariate. Multiple comparisons were performed using the Bonferroni post hoc test after ANOVA and ANCOVA. A Student’s *t*-test for paired samples was used to compare results on day 7 with results on day 30 within each group.

The Kruskal–Wallis non-parametric test was used to compare the groups in terms of the percentage of collagen (types I and III) and Gomori’s trichrome staining. Dunn’s test with Bonferroni adjustment was used for multiple comparisons between groups. The normality of the variables was assessed using the Kolmogorov–Smirnov test. Values of *p* < 0.05 were considered statistically significant. The data were analyzed using the IBM SPSS Statistics v.20.0 computer program, IBM Corp. (Armonk, NY, USA).

Post hoc analyses (i.e., pairwise comparisons performed after a global test, such as ANOVA or Kruskal–Wallis, indicate a significant difference) were conducted with multiple comparison corrections: Bonferroni adjustment following ANOVA and Dunn’s test with Bonferroni correction following the Kruskal–Wallis test. These corrections multiply the unadjusted *p*-values by the number of pairwise comparisons, which can result in adjusted *p*-values exceeding 1. In such cases, it is standard practice to report *p* = 1, as this is the maximum possible value for a *p*-value.

## 3. Results

Seventy rats participated in this study, all of which underwent experimental infarction. Thirteen animals (18.57%) died following this procedure. On the seventh day of the experiment, echocardiography was performed and 21 animals (36.8%) had an ejection fraction > 45%, so they were excluded from the study and euthanized.

The remaining thirty-six animals were randomly assigned to three study groups: one group received biomaterial implantation, one received sodium chloride (control), and one received neither. Thirteen animals died during the 30-day segment, leaving 23 animals for the final echocardiographic evaluation. In total, 26 animals (37%) died throughout the study period. The groups were divided as follows: control group (n = 7), WJ group (n = 8), and HAM group (n = 8). Following euthanasia, the hearts of all animals were dissected for histopathological analysis to determine the size of the AMI and the volume of collagen I and III deposits. Two hearts were excluded from the final analysis because they presented infarcts that were considered to be small under the microscope (WJG, n = 1; HAMG, n = 1), leaving 21 hearts for the final histopathological analysis.

### 3.1. Results of Cardiac Function Test

#### 3.1.1. Inter-Group Evaluation

Inter-group analysis on day seven post-MI revealed homogeneity among the CG, WJG, and HAMG, with no significant differences observed in LVEF (%), ESV (mL), or EDV (mL) (*p* = 0.601, *p* = 0.127, and *p* = 0.079, respectively). Similarly, on day thirty post-treatment, no statistically significant differences were found among the groups for the same parameters: LVEF (%) (*p* = 0.273), ESV (mL) (*p* = 0.865), and EDV (mL) (*p* = 0.838) (Table 1).

#### 3.1.2. Intra-Group Analysis

(A) Left Ventricular Ejection Fraction (LVEF %)

In the intra-group analysis from day seven to day thirty, LVEF (%) decreased from 36.2% to 33.4% in the CG (*p* = 0.331), increased from 36.6% to 39.7% in the WJG (*p* = 0.381), and rose from 34% to 36.8% in the HAMG (*p* = 0.140). However, none of these changes were statistically significant.

(B) End-Systolic Volume (ESV mL)

Regarding ESV (mL), an increase in volume was observed in all three groups by day thirty compared to day seven. In the CG, ESV increased from 0.165 ± 0.047 mL to 0.219 ± 0.082 mL (*p* = 0.097); in the WJG, from 0.107 ± 0.053 mL to 0.160 ± 0.089 mL (*p* = 0.053); and in the HAMG, from 0.155 ± 0.069 mL to 0.199 ± 0.063 mL (*p* = 0.159). None of these changes reached statistical significance.

(C) End-Diastolic Volume (EDV mL)

**Figure 4:**
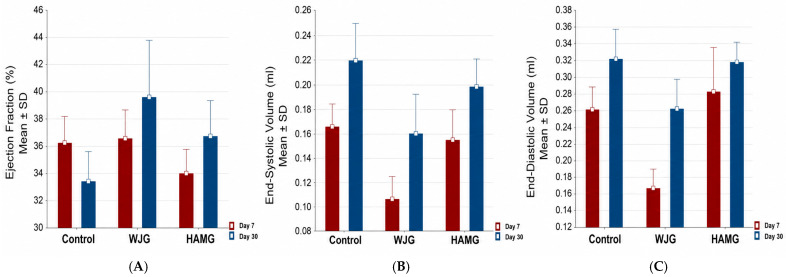
Compare echocardiographic results on day 7 and day 30 after myocardial infarction. (**A**) Ejection fraction (EF, %), (**B**) End-Systolic Volume (ESV, mL), (**C**) End-Diastolic Volume (EDV, mL). Results are expressed as mean ± standard error. Sample sizes: Control, (n = 7); WJG: Wharton’s Jelly group, (n = 8); and HAMG: Human Amniotic Membrane group, (n = 8).

Analysis of EDV (mL) also revealed increases in all three groups. In the CG, EDV rose from 0.263 ± 0.067 mL to 0.323 ± 0.091 mL (*p* = 0.112); in the WJG, from 0.167 ± 0.063 mL to 0.264 ± 0.098 mL—this increase reached statistical significance (*p* = 0.005); and in the HAMG, from 0.283 ± 0.148 mL to 0.319 ± 0.067 mL (*p* = 0.492) (Table 2 and Figure 4).

#### 3.1.3. Histopathological Analysis of Collagen Percentage

**Figure 5:**
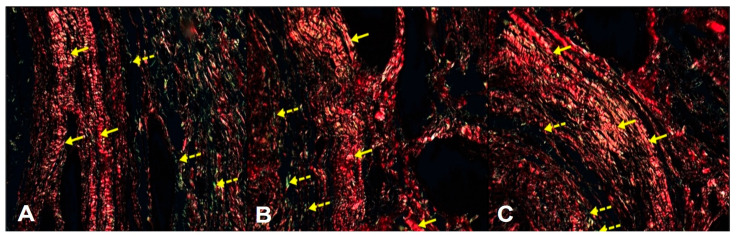
Infarct collagen content assessment after 30 days. A reduction in infarcted areas is observed in the treated groups. Picrosirius Red staining (400×, scale bar = 50 μm) reveals increased collagen deposition. (**A**) Control group (CG), (**B**) Wharton’s Jelly group, and (**C**) Human Amniotic Membrane group (HAMG). Type I collagen (red) and type III collagen (green) are indicated by solid and dotted yellow arrows, respectively. (**A**) control group; this group did not show an increase in collagen.

Analysis of collagen I and III deposition was performed on a sample of 21 hearts and is presented in 400× micrographs. Picrosirius Red staining highlights type I collagen in red and type III collagen in green, as indicated by solid and dashed yellow arrows, respectively (Figure 5).

Analysis under polarized light revealed that the scar areas in the control group (CG) had a predominance of type I collagen (97.3% ± 3.1%), followed by the Wharton’s jelly group (WJG, 90% ± 13.2%) and the human amniotic membrane group (HAMG, 85.2% ± 15.1%). However, these differences were not statistically significant (*p* = 0.74). Similarly, the percentages of type III collagen were 2.7% ± 3.1% for CG, 10% ± 13.2% for WJG, and 14.8% ± 15.1% for HAMG, with no statistically significant differences (*p* = 0.74) (Table 3).

#### 3.1.4. Histopathological Analysis of the Infarct Area

**Figure 6:**
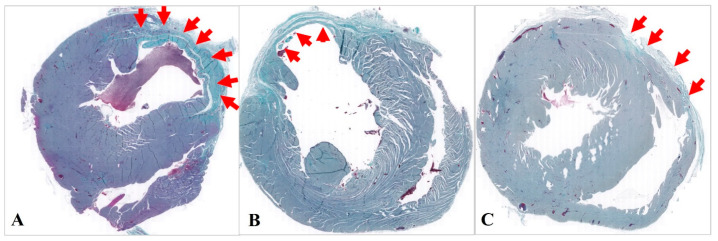
Histopathological analysis using Picrosirius Red and Gomori’s trichrome staining performed thirty days after experimental infarction. (**A**) Histological section of CG, (**B**) Histological section of WJG, and (**C**) Histological section of HAMG.

For infarct area analysis, Gomori’s trichrome-stained sections were used from 21 slides, with CG (n = 7), WJG (n = 7), and HAMG (n = 7). The infarct region was delineated based on the extent of collagen fiber staining, which predominates in the fibrotic area (Figure 6), and quantified by measuring the area (μm^2^).

The CG showed the largest scar area (18 × 10^6^ μm^2^), followed by the WJG (9 × 10^6^ μm^2^), while the HAMG presented the smallest scar area (8 × 10^6^ μm^2^). These differences were statistically significant (*p* = 0.008) (Table 4). Pairwise comparisons revealed a significant difference in infarct size between the CG (18 × 10^6^ μm^2^) and HAMG (8 × 10^6^ μm^2^) (*p* = 0.009).

**Figure 7:**
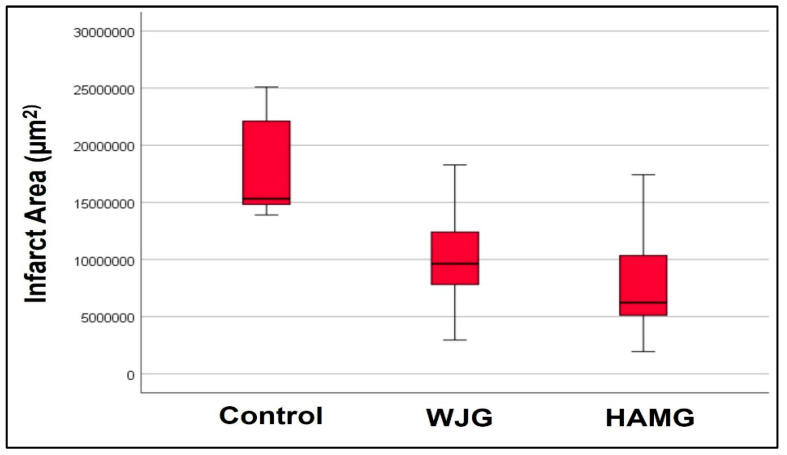
Infarct area content assessment after 30 days: Control group; Wharton’s Jelly group (WJG); and Human Amniotic Membrane group (HAMG).

These differences were statistically significant (*p* = 0.008). Pairwise comparisons revealed a significant difference in infarct size between the CG (18 × 10^6^ µm^2^) and HAMG (8 × 10^6^ µm^2^) (*p* = 0.009) (Table 4 and Table 5, Figure 7).

#### 3.1.5. Immunohistochemical Analysis

The markers used for the immunohistochemical analysis of the myocardial slides were Factor VIII, SMA (αActin), CD31, CD68, desmin, and sarcomeric actin. Table 6 shows the results obtained from the analysis of these markers.

#### 3.1.6. Factor VIII Expression

Immunohistochemical staining for Factor VIII, an indicator of angiogenesis and endothelial cell formation, revealed only subtle differences among the groups. The control group (CG) exhibited an average of 1.60 ± 1.23 positive cells, while the WJG showed a lower mean of 0.80 ± 1.39, and the HAMG had an intermediate mean of 1.01 ± 0.76. These numerical differences were not statistically significant (*p* = 0.240), suggesting that the tested biomaterials did not consistently influence neovascularization.

#### 3.1.7. α-Actin (SMA)

α-Actinin plays a fundamental role in cytoskeletal organization and in the contractile function of smooth muscle cells, and it is widely used as a marker of myofibroblast presence and fibrotic events related to the *ACTA2* gene. Analysis of α-actin expression showed a mean value of 2.23 ± 1.59 in the control group, while the WJG presented a higher mean of 5.53 ± 7.60 and the HAMG exhibited an intermediate mean of 3.51 ± 2.34. Although a tendency toward higher α-actin expression was observed in the WJG, no statistically significant differences were detected among the groups (*p* = 0.734), indicating that the evaluated treatments did not induce significant changes in myofibroblast activation markers.

#### 3.1.8. CD31 Expression

Analysis of CD31, a marker for new blood vessel formation, revealed slight variations among groups but no statistically significant differences (*p* = 0.857). The control group (CG) had an average of 0.36 ± 0.43, the WJG showed a higher mean of 0.76 ± 1.55, and the HAMG had an average similar to that of CG at 0.31 ± 0.27. Although the WJG tended to show higher CD31 levels, these differences were not robust, suggesting that the treatments did not have a significant impact on angiogenesis.

#### 3.1.9. CD68 Expression

Immunohistochemical analysis for CD68, a marker for macrophage infiltration, revealed no significant differences among the groups four weeks after MI induction. The number of CD68-positive cells was similar across all treatment groups. The control group (CG) had an average of 0.19 ± 0.22, the WJG showed a higher mean of 0.48 ± 0.65, and the HAMG had an average similar to the WJG at 0.40 ± 0.69 (*p* = 0.877), indicating that the biomaterials did not significantly modulate the inflammatory response in terms of macrophage infiltration.

#### 3.1.10. Desmin Expression

Desmin, a marker associated with muscle integrity and regeneration, showed numerical differences among groups but without statistical significance (*p* = 0.251). The control group (CG) averaged 0.53 ± 0.67, the WJG averaged 0.76 ± 0.98, and the HAMG had the highest mean at 2.41 ± 3.96. Although the HAMG’s higher value suggests a possible enhanced contribution to structural regeneration, the variability and lack of significance indicate that the biomaterials did not consistently affect Desmin expression.

#### 3.1.11. Sarcomeric α-Actin

Evaluation of sarcomeric α-Actin, a marker for muscle regeneration and structural integrity, revealed no significant differences among the groups (*p* = 0.577). The control group (CG) had an average of 2.54 ± 2.21, the WJG showed similar values at 2.42 ± 2.81, and the HAMG exhibited a slightly higher mean of 3.41 ± 2.41. Although there was a trend toward higher α-Actin expression in the HAMG, the differences were not statistically significant, indicating that the treatments did not significantly alter myocardial structural regeneration.

## 4. Discussion

The present study investigated the effects of decellularized human amniotic membrane (HAM) and Wharton’s jelly (WJ) implantation on cardiac remodeling after myocardial infarction in rats with established left ventricular dysfunction. Although no significant functional recovery was observed after 30 days, HAMG implantation led to a significant reduction in fibrotic area compared to the control group, suggesting histological modulation of post-infarction remodeling.

The reduction in scar area observed in the HAMG supports previous evidence that this biomaterial exerts anti-fibrotic and anti-inflammatory effects, possibly through its native bioactive components, even after decellularization [8,10,24]. Decellularized HAM has been shown to provide a favorable microenvironment that supports tissue repair, modulating fibroblast activity and attenuating excessive collagen type I deposition [13,25]. In this study, the HAMG exhibited smaller infarcted areas and a tendency toward greater organization of collagen fibers, although quantitative collagen type I/III ratios did not reach significance. Such structural effects without measurable functional improvement are consistent with early-stage remodeling, which may require longer follow-up to manifest in echocardiographic parameters [15,26]. Wharton’s jelly, on the other hand, did not demonstrate comparable benefits. Its ECM, rich in glycosaminoglycans and hyaluronic acid, has been associated with enhanced angiogenesis in other models [9,11], but the short follow-up and absence of cell seeding may have limited its reparative potential.

One key aspect to highlight is the rationale behind choosing decellularized perinatal tissues over native ones. This preference is justified by the inherent non-immunogenic properties of native perinatal tissues, eliminating the need for their use [27]. We acknowledge this important clarification. The use of decellularization in this context was not intended to prevent immune rejection, but rather to achieve a reproducible, cell-free scaffold with reduced donor variability and a controlled extracellular matrix structure. Decellularization also facilitates future integration with cells or growth factors in combinatorial strategies [12,14,16].

Recent advances have demonstrated that biomaterials alone often yield modest improvements, whereas hybrid strategies combining decellularized ECM with mesenchymal stem cells (MSCs) or proangiogenic molecules enhance myocardial regeneration [15,16,17]. Such combinations promote cellular infiltration, angiogenesis, and contractile tissue organization. Therefore, the histological improvement seen with HAM alone may represent the foundational step toward a future bioactive hybrid scaffold for myocardial repair. In agreement with [4,5], our findings underscore that the greatest translational value of ECM scaffolds lies not in their isolated effects but in their capacity to serve as a biologically compatible platform for advanced regenerative therapies.

The absence of statistically significant differences in immunohistochemical markers of angiogenesis and myocardial regeneration (Factor VIII, CD31, desmin, sarcomeric actin) warrants specific discussion. Several convergent factors may explain these findings. First, both HAM and WJ were implanted as cell-free scaffolds, without the addition of exogenous stem cells or growth factors. In the absence of such stimuli, the magnitude of angiogenic and myogenic signaling elicited by the decellularized ECM alone may be insufficient to produce detectable immunohistochemical differences within a 30-day observation window. Second, the relatively short follow-up period may not have allowed adequate time for downstream regenerative cascades to manifest at the histological level. Third, the modest sample size and biological variability inherent to experimental infarction models may have reduced statistical power for these molecular endpoints. Collectively, these observations are consistent with the hypothesis that the primary mechanism of action of HAM in this model is anti-fibrotic structural modulation, rather than direct stimulation of neovascularization or cardiomyocyte regeneration. Future studies incorporating proangiogenic molecules such as VEGF or bFGF, or combinatorial cell-seeded scaffolds, will be necessary to assess whether the structural improvements observed here can be translated into measurable regenerative responses.

## 5. Conclusions

In summary, epicardial implantation of decellularized human amniotic membrane (HAM) reduced myocardial scar area in rats with post-infarction ventricular dysfunction, demonstrating a predominantly anti-fibrotic structural effect. Although no significant echocardiographic improvement was observed within 30 days, the findings suggest that attenuation of fibrosis may precede functional recovery during early remodeling. Decellularized Wharton’s jelly did not show similar benefits, and immunohistochemical markers of angiogenesis and regeneration remained unchanged among groups. Overall, these results support the potential of decellularized perinatal ECM scaffolds as biocompatible platforms for post-infarction cardiac repair and as promising carriers for future combinatorial regenerative therapies.

## 6. Study Limitations

The main limitation of this study was the small number of human perinatal tissue donors (n = 2), lack of quantitative validation of the decellularization process. In addition, the 30-day follow-up may have been insufficient to detect long-term functional improvements. Immunohistochemical analyses were restricted to general structural and inflammatory markers, without evaluation of cytokines or molecular mediators involved in cardiac remodeling. Furthermore, serial measurements of cardiac biomarkers (cTnI, cTnT, CK-MB, and BNP) were not performed, limiting the characterization of the acute injury phase and its relationship with tissue remodeling. Nevertheless, this study is valuable as it is one of the first clinical trials in Brazil to use Wharton’s jelly and amniotic membrane for cardiac tissue regeneration following acute myocardial infarction.

## 7. Future Perspectives

Future studies should include quantitative ECM characterization, longer follow-up periods, and the incorporation of cellular or molecular factors to improve tissue repair. Serial analysis of cardiac biomarkers and inflammatory cytokines may better correlate acute myocardial injury with remodeling and biomaterial-mediated regeneration. In addition, the use of stem cells, growth factors such as VEGF and TGF-β, and multilayered recellularized constructs may enhance functional recovery. Future investigations should also include appropriate comparative control groups, including next-generation pharmacological therapies for cardiac regeneration, to better define the therapeutic benefits of these bioengineering strategies.

## Figures and Tables

**Table 1:** Echocardiographic evaluations: representative flow parameters from each group before 7- and 30-day treatment.

Variable	Groups	Mean ± SD	* *p* Compared Groups
**LVEF (%)** **Day 7**	Control (n = 7)	36.2 ± 5.3	0.601
	WJG (n = 8)	36.6 ± 6.0	
	HAMG (n = 8)	34.0 ± 5.0	
**ESV (mL)** **Day 7**	Control (n = 7)	0.165 ± 0.047	0.127
	WJG (n = 8)	0.107 ± 0.053	
	HAMG (n = 8)	0.155 ± 0.069	
**EDV (mL)** **Day 7**	Control (n = 7)	0.263 ± 0.067	0.079
	WJG (n = 8)	0.167 ± 0.063	
	HAMG (n = 8)	0.283 ± 0.148	
**LVEF (%)** **Day 30**	Control (n = 7)	33.4 ± 5.8	0.273
	WJG (n = 8)	39.7 ± 11.7	
	HAMG (n = 8)	36.8 ± 7.4	
**ESV (mL)** **Day 30**	Control (n = 7)	0.219 ± 0.082	0.865
	WJG (n = 8)	0.160 ± 0.089	
	HAMG (n = 8)	0.199 ± 0.063	
**EDV (mL)** **Day 30**	Control (n = 7)	0.323 ± 0.091	0.838
	WJG (n = 8)	0.264 ± 0.098	
	HAMG (n = 8)	0.319 ± 0.067	

Day 7: Post-infarction echocardiographic evaluation; Day 30: Post-treatment echocardiographic evaluation; LVEF: Left Ventricle Ejection Fraction; ESV: End-Systolic Volume; EDV: End-Diastolic Volume; WJG: Wharton’s Jelly group; HAMG: Human Amniotic Membrane group; * Student’s *t*-test for paired samples; the data are presented as mean ± standard deviation. *p*-values indicate statistically significant differences (*p* < 0.05).

**Table 2:** Assessing cardiac function using conventional echocardiography was performed across diverse groups in the present study.

Variable	Groups	Day 7 Mean ± SD	Day 30 Mean ± SD	* *p* Compared Groups
**LVEF (%)**	Control (n = 7)	36.2 ± 5.3	33.4 ± 5.8	0.331
	WJG (n = 8)	36.6 ± 6.0	39.7 ± 11.7	0.381
	HAMG (n = 8)	34 ± 5.0	36.8 ± 7.4	0.140
**ESV (mL)**	Control (n = 7)	0.165 ± 0.047	0.219 ± 0.082	0.097
	WJG (n = 8)	0.107 ± 0.053	0.160 ± 0.089	0.053
	HAMG (n = 8)	0.155 ± 0.069	0.199 ± 0.063	0.159
**EDV (mL)**	Control (n = 7)	0.263 ± 0.067	0.323 ± 0.091	0.112
	WJG (n = 8)	0.167 ± 0.063	0.264 ± 0.098	0.005
	HAMG (n = 8)	0.283 ± 0.148	0.319 ± 0.067	0.492

Day 7 and Day 30: Post-infarction echocardiographic evaluation; LVEF: Left Ventricle Ejection Fraction; ESV: End-Systolic Volume; EDV: End-Diastolic Volume; WJG: Wharton’s Jelly group; HAMG: Human Amniotic Membrane group. Data are shown as mean ± standard deviation. Bonferroni post hoc test. Values of *p* < 0.05 denote statistical significance. * The ejection fraction are values are expressed as percentage (%).

**Table 3:** Inter-group analysis of collagen types at 30 days post-infarction.

Variable	Groups	Mean ± SD	* *p* Compared Groups
Collagen I (%)	Control (n = 7)	97.3 ± 3.1	-
	WJG (n = 7)	90 ± 13.2	-
	HAMG (n = 7)	85.2 ± 15.1	0.074
Collagen III (%)	Control (n = 7)	2.7 ± 3.1	-
	WJG (n = 7)	10 ± 13.2	-
	HAMG (n = 7)	14.8 ± 15.1	0.074

WJG: Wharton’s Jelly group; HAMG: Human Amniotic Membrane group; * Bonferroni post hoc test. Values of *p* < 0.05 denote statistical significance. The *p*-values for collagen types I and III are identical, as their combined percentage totals 100%.

**Table 4:** Inter-group analysis of infarct area (µm^2^) at 30 days post-infarction.

Variable	Groups	Mean ± SD	* *p* (Compared Groups)
Infarct Area (µm^2^)	Control (n = 7)	18,306,347 ± 4,795,936	-
	WJG (n = 7)	9,675,381 ± 4,877,956	-
	HAMG (n = 7)	8,077,347 ± 5,134,477	0.008

Values expressed in µm^2^. WJG: Wharton’s Jelly group; HAMG: Human Amniotic Membrane group; * Values of *p* < 0.05 denote statistical significance.

**Table 5:** Two-by-two comparison of infarct area (µm^2^), after 30 days of follow-up.

Groups Comparison	* *p* (Compared Groups)
Control vs. WJG	0.060
Control vs. HAMG	0.009

Values expressed in µm^2^. WJG: Wharton’s Jelly group Human Amniotic Membrane group (HAMG); * Values of *p* < 0.05 denote statistical significance.

**Table 6:** The markers used for immunohistochemical analysis of myocardial slides.

Variable	Groups	Mean ± SD	*p* Compared Groups
**Factor VIII (%)**	Control (n = 7)	1.60 ± 1.23	-
	WJG (n = 7)	0.80 ± 1.39	-
	HAMG (n = 7)	1.01 ± 0.76	0.240
**α-Actin (%)**	Control (n = 7)	2.23 ± 1.59	-
	WJG (n = 7)	5.53 ± 7.60	-
	HAMG (n = 7)	3.51 ± 2.34	0.734
**CD31 (%)**	Control (n = 7)	0.36 ± 0.43	-
	WJG (n = 7)	0.76 ± 1.55	-
	HAMG (n = 7)	0.31 ± 0.27	0.857
**CD68 (%)**	Control (n = 7)	0.19 ± 0.422	-
	WJG (n = 7)	0.48 ± 0.65	-
	HAMG (n = 7)	0.40 ± 0.69	0.877
**Desmin (%)**	Control (n = 7)	0.53 ± 0.67	-
	WJG (n = 7)	0.76 ± 0.98	-
	HAMG (n = 7)	2.41 ± 3.96	0.251
**Sarcomeric Actin (%)**	Control (n = 7)	2.54 ± 2.21	-
	WJG (n = 7)	2.42 ± 2.81	-
	HAMG (n = 7)	3.41 ± 2.41	0.577

WJG: Wharton’s Jelly group; HAMG: Human Amniotic Membrane group; Values of *p* < 0.05 denote statistical significance.

## Data Availability

The original contributions presented in this study are included in the article. Further inquiries can be directed to the corresponding author.
